# Exploring the critical role of PANoptosis in the pathogenesis of intervertebral disc degeneration: mechanisms and potential therapeutic targets

**DOI:** 10.3389/fcell.2025.1611936

**Published:** 2025-06-19

**Authors:** Kaisheng Zhou, Mingyang Zou, Shaobo Wu, Wei Song, Hao Dong, Yanbo Dong, Haihong Zhang

**Affiliations:** ^1^ Department of Orthopaedics, Lanzhou University Second Hospital, Lanzhou, China; ^2^ Orthopaedics Key Laboratory of Gansu Province, Lanzhou, China

**Keywords:** PANoptosis, programmed cell death, nucleus pulposus cell, intervertebral disc degeneration, mechanism

## Abstract

Intervertebral disc degeneration (IVDD), a leading cause of chronic low back pain, imposes a significant global health burden due to its association with aging, inflammation, and mechanical stress. Emerging evidence highlights programmed cell death (PCD) as a pivotal driver of IVDD progression. PANoptosis, a novel integrated cell death mechanism combining pyroptosis, apoptosis, and necroptosis, has recently gained attention for its role in amplifying inflammatory responses and accelerating disc degeneration. This review synthesizes current knowledge on PANoptosis in nucleus pulposus cells (NPCs), emphasizing its regulatory crosstalk via multiprotein complexes and signaling pathways such as RIPK, caspase activation, and gasdermin-mediated membrane permeabilization. Key triggers, including oxidative stress, cytokine dysregulation, and mechanical compression, exacerbate PANoptosis, leading to NPC loss and extracellular matrix degradation. While therapeutic strategies targeting PANoptosis-related molecules show promise in preclinical studies, clinical translation remains limited. Elucidating the interplay between PANoptosis and other pathological pathways could unveil novel biomarkers and therapeutic targets. This review underscores PANoptosis as a critical axis in IVDD pathogenesis and advocates for multidisciplinary approaches to bridge mechanistic insights into effective clinical interventions.

## 1 Introduction

Intervertebral disc degeneration (IVDD), recognized as the most prevalent chronic orthopedic disorder and a primary contributor to chronic low back pain, imposes substantial global disease burden (GBD 2021 Neck Pain Collaborators, 2024). This condition profoundly diminishes individuals’ quality of life while generating significant socioeconomic impacts (Maher et al., 2017; Hartvigsen et al., 2018). Age-related degenerative changes in disc structure and function, leading to the death of intervertebral disc cells, which is pivotal in the pathological process of IVDD (Wang et al., 2016). Numerous genetic and environmental risk factors, such as smoking, aging, trauma, and occupational exposure, are recognized as contributors to IVDD (Han, 2020; Feng et al., 2016; Lu et al., 2022). Since then, research has revealed that the pathogenesis of IVDD includes nucleus pulposus cells (NPCs) senescence and apoptosis, inflammatory stimulation, extracellular matrix (ECM) degradation, oxidative stress, and other contributing factors (Feng et al., 2016; Feng et al., 2017; Liu et al., 2018). However, the pathogenesis of IVDD remains largely unknown. Research indicates that the pattern of NPC injury in IVDD closely resembles programmed cell death (PCD). Currently, it has been established that the occurrence and progression of IVDD are regulated by various modes of cell death (Liao et al., 2019; Vergroesen et al., 2015; Chan et al., 2016). Therefore, clarifying the mechanisms of different cytokines and cell death modes associated with IVDD is crucial for understanding its pathogenesis.

PCD is a highly regulated biological process that is essential for maintaining tissue homeostasis and eliminating damaged or unnecessary cells (Yuan and Ofengeim, 2024; Tower, 2015). Over the past few decades, various new forms of non-apoptotic PCD have been discovered, including cuproptosis, autophagy-dependent cell death, disulfidptosis, alkaliptosis, ferroptosis, lysosome-dependent cell death, necroptosis, netotic cell death, oxeiptosis, parthanatos, and pyroptosis (Chen et al., 2023; Zhao et al., 2022; Wu et al., 2024; Andrabi et al., 2008; Fink and Cookson, 2005; Yang and Stockwell, 2016; Lin et al., 2025; Lu et al., 2025; Kulkarni and Hardwick, 2023). Currently, the most common modes of cell death are apoptosis, necroptosis, and autophagy (Yuan and Ofengeim, 2024; Quan et al., 2020; Cazzanelli and Wuertz-Kozak, 2020). Abnormal PCD is closely linked to the development of various diseases, including cancer (Guo et al., 2024), autoimmune diseases (Liu et al., 2024), and neurodegenerative disorders (Yu et al., 2017). Additionally, PCD plays a significant role in the development of IVDD. Research on the relationship between PCD and IVDD has primarily focused on cell death within the intervertebral disc. particularly in the nucleus pulposus, located at the center of the disc, which is a key feature (Lu et al., 2025; Li et al., 2017; Cao X. et al., 2022). Although the precise relationship between PCD and IVDD is still unclear, it can be posited that PCD may contribute to the progression of IVDD by affecting cell survival.

**FIGURE 1:**
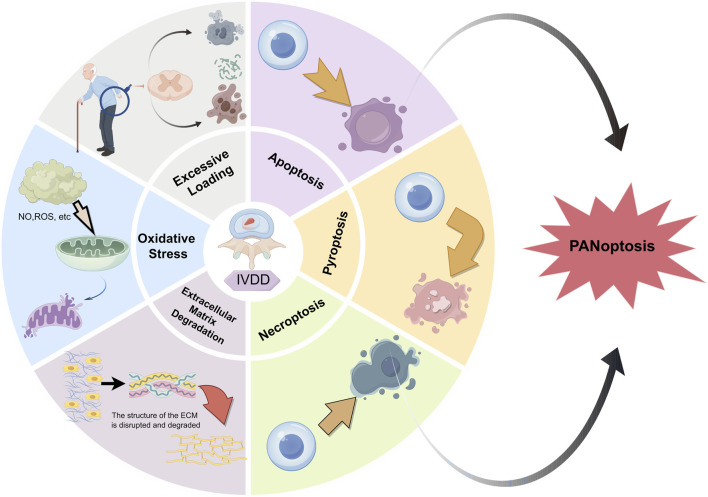
Summary of possible causes of IVDD. The potential possible causes to IVDD are presented as follows: excessive stress, oxidative damage, extracellular matrix degeneration, and PANoptosis (with P signifying pyroptosis, A denoting apoptosis, and N representing necroptosis).

Although the relationship between PCD and the occurrence of IVDD is intimate, the occurrence process cannot be perfectly explained by a single theory. Our recent research indicates that multiple key genes in various PCD pathways are involved in the occurrence and development of IVDD (Zou et al., 2025). Hence, the joint regulation of multiple PCD pathways might be a superior strategy for the treatment of IVDD. For instance, the combined regulation of apoptosis and necroptosis can significantly enhance the survival of NPCs (Chen et al., 2017a; Chen et al., 2018). Based on this, our focus has shifted to PANoptosis (P represents pyroptosis, A represents apoptosis, and N represents necroptosis), a novel form of cell death that was first proposed by Malireddi et al. (2019); Samir et al. (2020). It is characterized by the concurrent occurrence of necroptosis, apoptosis, and pyroptosis, but cannot be fully explained by any one of these mechanisms alone (Samir et al., 2020; Malireddi et al., 2019; Christgen et al., 2022). PANoptosis is an evolving field of study. As research advances, it is expected to be increasingly refined. Thus, we conducted this review to investigate the role of PANoptosis in nucleus pulposus cell death during IVDD, as shown in Figure 1.

## 2 Mechanisms and crosstalk events in PANoptosis

### 2.1 Apoptosis

Apoptosis is a complex process of PCD that involves various molecules and pathways, which can generally be categorized into intrinsic and extrinsic pathways. They are also called the mitochondrial pathway and the death receptor pathway, respectively (Bovin and Bendtzen, 1999). However, these two apoptotic pathways are interconnected via the mitochondria and converge at a stage referred to as the apoptotic execution phase. Intrinsic pathways are essential for cellular responses to various stimuli, including growth factors, hypoxia, oxidative stress, and DNA damage (Mirza-Aghazadeh-Attari et al., 2019). These pathways activate downstream B cell lymphoma (BCL) family proteins, encompassing both antiapoptotic members like Bcl-2, Bcl-XL, Bcl-9, and MCL-1, and proapoptotic members such as Bax and Bak. The culmination of these interactions promotes apoptosis primarily by inhibiting antiapoptotic factors, thereby shifting the balance toward cell death (Jeng et al., 2010; Kashyap et al., 2021; Garner et al., 2016; Westphal et al., 2014).

Exogenous pathways involve the activation of death receptor families, with the most classical being the tumor necrosis factor (TNF) and tumor necrosis factor receptor (TNFR) signaling pathways, as well as CD95 (also known as Fas or APO-1) (Pistritto et al., 2016). The TNFR superfamily consists of two primary receptors: TNFR-1 and TNFR-2. TNFR-1 features a death domain characterized by a homologous amino acid sequence, which is crucial for apoptotic signaling. Apoptosis is initiated when the death domain of TNFR-1 interacts with the TNF receptor associated death domain (TRADD) binding protein. This interaction recruits the Fas associated death domain (FADD) protein, facilitating the assembly and activation of procaspase-8, which leads to the formation of the death inducing signaling complex (DISC). FADD, in conjunction with procaspase-8, forms the DISC, resulting in the catalytic activation of procaspase-8. Activated caspase-8 then propagates the apoptotic signal by cleaving and activating downstream effector caspases, such as caspase-3 and caspase-7, ultimately orchestrating the apoptotic process (Green, 2022; Kurosaka et al., 2003; Lossi, 2022).

### 2.2 Pyroptosis

Unlike apoptosis and simple cell necrosis, cellular pyroptosis is inherently pro-inflammatory. It is characterized by a robust inflammatory response mediated by the activation of cytosolic multiprotein complexes known as inflammasomes. Pyroptosis represents a lytic and inflammatory form of PCD that is executed by pore-forming proteins called gasdermins. The gasdermin family includes GSDMA, GSDMB, GSDMC, GSDMD, GSDME, and GSDMF (PJVK/DFNB59) (Broz et al., 2020).

When the inflammasome is activated, an enzyme known as caspase-1 is recruited and subsequently activated. Once activated, caspase-1 cleaves gasdermin D, a key protein involved in pyroptosis. Additionally, active caspase-1 facilitates the recruitment of effector caspase-3, which cleaves gasdermin D/E (GSDMD/E) at its C-terminal region (GSDMD/EC), thereby stimulating the release of the N-terminal portion (GSDMD/E-N). The cleavage of gasdermin D results in the formation of pores in the cell membrane, leading to cell membrane rupture. Consequently, inflammatory factors, including interleukins (IL)-1β and IL-18, are released from the cells (Yu et al., 2021; Liston and Masters, 2017).

### 2.3 Necroptosis

Necroptosis is characterized by features of both necrosis and apoptosis, as its definition suggests. It is triggered by death receptor ligands, such as TNFR1, TNFR2, and ligands like TNF-α and Fas ligand (FasL), which inhibit the apoptotic pathway (Linkermann and Green, 2014; Martens et al., 2021). RIPK1 is an upstream regulator and serves as a crucial signaling node in various signal transduction pathways, actively regulating the balance between gene activation and cell death, including apoptosis and necroptosis (Annibaldi and Meier, 2018).

Downstream of the aforementioned receptors, active RIPK1 is recruited into an oligomeric complex that includes FADD, caspase-8, and caspase-10 (Tenev et al., 2011; Feoktistova et al., 2011). In the absence of caspase-8 activity, RIPK1 recruits and phosphorylates RIPK3, resulting in the formation of a complex known as the pro-necrotic RIP1-RIP3 complex (Cho et al., 2009; Li et al., 2012). Subsequently, the RIPK1/RIPK3 complex recruits and phosphorylates MLKL (mixed lineage kinase domain like protein), leading to the formation of the necrosome and the execution of necroptosis (Zhao et al., 2012; Murphy et al., 2013).

### 2.4 Crosstalk and regulation in PANoptosis

Studies have emphasized that pyroptosis, apoptosis, and necroptosis are not distinct and independent pathways as previously thought. For example, a series of studies have demonstrated that functional alteration of receptor-interacting serine/threonine protein kinases (RIPKs) can induce pyroptosis, apoptosis and necroptosis simultaneously (Kesavardhana et al., 2020; Kaiser et al., 2014; Newton et al., 2016). Subsequently, Malireddi et al. demonstrated the existence of a single cell death-inducing complex that controls all three pathways (Malireddi et al., 2019; Malireddi et al., 2020). They can occur simultaneously within the same cell and are mediated by multiprotein complexes known as the PANoptosome (Samir et al., 2020; Christgen et al., 2020). RIPK1, apoptosis-associated speck-like protein, RIPK3, CASP6, Z-DNA-binding protein 1 (ZBP1) and CASP1 were identified as components of PANoptosome (Malireddi et al., 2020; Christgen et al., 2020). This complex encompasses key features of all three forms of PCD but cannot be fully explained by any of them alone (Malireddi et al., 2020; Gurung et al., 2016; Malireddi et al., 2018; Ma et al., 2025). Specifically, there are currently four confirmed PANoptosome structures, namely, ZBP1-PANoptosome, RIPK1-PANoptosome, AIM2-PANoptosome, and NLRP12-PANoptosome. Their core components are listed in Table 1.

**TABLE 1:** The specific composition of four different PANoptosome complexes.

The type of PANoptosome complex	Specific composition
ZBP1-PANoptosome	ZBP1, RIPK3, NLRP3, RIPK1, ASC, Casp8, Casp6, Casp1
RIPK1-PANoptosome	RIPK1, RIPK3, NLRP3, Casp8, ASC, Casp1
AIM2-PANoptosome	AIM2, ZBP1, ASC, RIPK3, Casp1, RIPK1, FADD, Casp8
NLRP12-PANoptosome	NLRP12, RIPK3, NLRP3, ASC, RIPK1, Casp8, Casp1

Therefore, we can recognize that PANoptosis is a constantly evolving and dynamic field, rather than a simple aggregation of the three types of PCD (Table 2). Herein, we systematically delineate the regulatory mechanisms and molecular interplay between PANoptosis and the three PCD pathways.

**TABLE 2:** The basic difference between apoptosis, pyroptosis, necroptosis, and PANoptosis.

Feature	Apoptosis	Pyroptosis	Necroptosis	PANoptosis
Core pathway	Intrinsic/Extrinsic	Inflammasome-GSDMD	RIPK1/RIPK3/MLKL	PANoptosome
Key molecules	Caspases-3/8, BCL-2	Caspase-1, GSDMD/E	RIPK1, MLKL	RIPK1/3, Caspases-1/3/8
Membrane fate	Blebbing (intact)	Pore rupture (lytic)	Rupture (necrotic)	Combined lytic rupture
Inflammation	Non-inflammatory	Strongly inflammatory	Delayed inflammatory	Hyper-inflammatory
Trigger	DNA damage, TNF/FasL	Pathogens, DAMPs	Apoptosis inhibition	Infection, tissue damage
Complex	DISC	Inflammasome	Necrosome	PANoptosome

Intrinsic/Extrinsic, Mitochondrial/death receptor pathways; GSDMD/E, Gasdermin D/E (pore-forming proteins); RIPK1/RIPK3/MLKL, Receptor-interacting protein kinase 1/3, mixed lineage kinase domain-like protein; BCL-2, B-cell lymphoma-2 family proteins; DISC, Death-inducing signaling complex; DAMPs, Damage-associated molecular patterns.

The interplay between PANoptosis and apoptosis is particularly noteworthy, as both processes share common signaling pathways, including those involving caspases and receptor interacting protein kinases (RIPKs) (Tsuchiya et al., 2019). Research indicates that PANoptosis can be initiated through caspase activation, a hallmark of apoptosis; however, it diverges by incorporating elements of inflammatory cell death, thereby enhancing the immune response to cellular stress or damage (Kesavardhana et al., 2020). For instance, the activation of RIPK3 in PANoptosis can lead to the formation of PANoptosomes, complexes that facilitate cell death while simultaneously promoting inflammation through the release of pro-inflammatory cytokines (Man and Kanneganti, 2016). This cross regulation highlights a complex network in which apoptotic signals can either promote or inhibit PANoptosis, depending on the cellular context and the presence of specific stimuli.

The relationship between PANoptosis and pyroptosis is characterized by a shared reliance on inflammatory pathways; however, they exhibit distinct mechanisms and outcomes (Cai et al., 2023). Pyroptosis is typically driven by the activation of caspase-1, leading to the formation of gasdermin pores in the cell membrane. This process results in cell lysis and the release of pro-inflammatory cytokines such as IL-1β and IL-18 (He et al., 2015). In contrast, PANoptosis encompasses a broader spectrum of cell death modalities, including pyroptosis, while also integrating aspects of apoptosis and necroptosis. The interplay between these pathways is significant in the context of various diseases (Liu et al., 2016; Shi et al., 2014).

**FIGURE 2:**
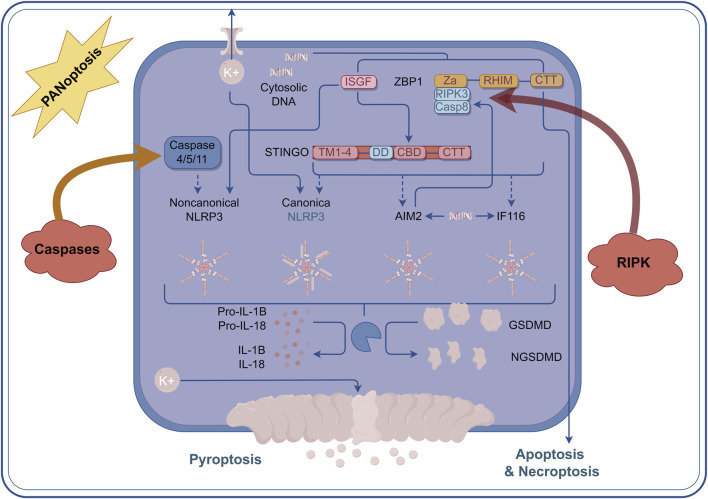
Crosstalk events in the mechanism of PANoptosis. Within the context of PANoptosis, multiple regulatory mechanisms are primarily achieved through the multiprotein complex PANoptosome. The Caspase family and the RIPK family play the most crucial roles. Under the guidance of the PANoptosome, a large number of crosstalk events occur, and the core elements of these events comprise the essential proteins involved in apoptosis, pyroptosis, and necroptosis.

PANoptosis is a rapid and efficient mode of cell death, typically associated with a strong inflammatory response that quickly eliminates damaged cells in the event of pathogen infection or cellular injury. In contrast, necroptosis is a relatively slow form of cell death, characterized by the rupture of the cell membrane, which leads to the release of cellular contents and subsequently triggers the inflammatory response in surrounding tissues (Choi et al., 2023). The interaction between these two forms of cell death is highly complex. Research indicates that the formation of the necrosome may inhibit the occurrence of PANoptosis, while conversely, the activation of PANoptosis may also influence necrosome formation (Han et al., 2011). Studies have shown that PANoptosis induces cell death through the interaction of multiple signaling pathways. For instance, intracellular inflammasomes can promote PANoptosis by activating caspase-8, while necrosomes facilitate cell death through a necrotic process mediated by MLKL (Davies et al., 2018). In addition, the NF-κB signaling pathway plays a crucial role in both cell death mechanisms by regulating the expression of pro-inflammatory factors and promoting the occurrence of cell death (Rodriguez et al., 2016). And NLRP12 was identified as a key node for the regulation of PANoptosis and was highly correlated with the regulation of inflammatosomes in IVDD (Navuluri et al., 2025). Here we summarized the Crosstalk events in the mechanism of PANoptosis (Figure 2).

## 3 Molecular mechanisms of PANoptosis in the nucleus pulposus cells

### 3.1 Apoptosis in NPCs

As classical apoptotic pathways, the death receptor, mitochondrial, and endoplasmic reticulum stress (ERS) pathways have been confirmed to be involved in the occurrence and development of IVDD (Heyde et al., 2006). During IVDD, rupture or inflammation of the annulus fibrosus induces nucleus pulposus (NP) cell apoptosis by promoting the production of various inflammatory mediators, including reactive oxygen species (ROS), interleukins (IL), nitric oxide (NO), and matrix degrading enzymes (Kang et al., 1995).

The Fas pathway may contribute to IVDD. Sun et al. demonstrated that the expression level of Fas in degraded NPC was significantly higher than in normal cells, suggesting its role in promoting NPC apoptosis (Sun et al., 2013). Wang et al. found that Fas receptor (FasR) expression and apoptosis in endplate cells were significantly elevated in degenerated discs compared to non-degenerated discs, indicating that Fas mediated apoptosis may occur in endplate cells (Wang F. et al., 2011). Liu et al. confirmed that Fas ligand (FasL) expression is reduced in degenerated discs, which plays a crucial role in maintaining immune privilege (Liu et al., 2013). The above studies suggest that Fas may have a dual role in regulating NPC apoptosis, and the specific mechanisms require further investigation.

Cytokines such as IL-1β and TNF-α exacerbate the inflammatory process and are considered key mediators of IVDD. Studies have shown that exposure of NPCs to elevated levels of IL-1β significantly increases the production of IL-8 and IL-6, as well as upregulating inflammatory mediators including prostaglandin E2, cyclooxygenase-2, and TNF-α (Jimbo et al., 2005; Jia et al., 2020; Wang et al., 2019; Jin et al., 2019; Fang and Jiang, 2016). As a pleiotropic cytokine, the expression of TNF-α is upregulated in response to mechanical loading (Wang et al., 2007). Moreover, it has been confirmed that TNF-α induces the secretion of inflammatory mediators such as IL-6, NO, and PGE2 in nucleus pulposus cells of patients with IVDD (Gabr et al., 2011). It has also been confirmed that the levels of CCL3, CCL20, CXCL2, and CXCL5 are significantly elevated in intervertebral disc cells following TNF-α stimulation (Liu et al., 2015). Additionally, oxidative damage (Han et al., 2019) and matrix metalloproteinases (MMPs) (Hiyama et al., 2010) have been shown to be associated with nucleus pulposus cell apoptosis and IVDD.

**FIGURE 3:**
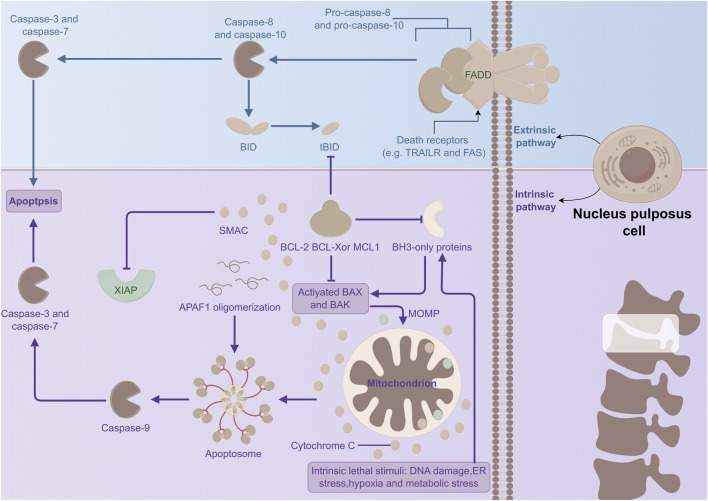
Mechanism of apoptosis in NPCs. The extrinsic pathway (namely, the death receptor pathway) and the intrinsic pathway (i.e., the mitochondrial pathway) trigger apoptosis. They jointly generate the apoptotic protein complex CASP, which functions as the essential executor of apoptosis and induces apoptosis.

In conclusion, various inflammatory mediators and multiple signaling pathways—including the ERS response pathway, the mitochondrial pathway, and the death receptor pathway—are implicated in NPC apoptosis, which is associated with IVDD (Figure 3). Currently, the molecular mechanisms underlying these signaling pathways and their interactions remain incompletely understood and warrant further investigation. Therapeutics developed from this understanding may offer novel approaches for the diagnosis and early treatment of IVDD.

### 3.2 Necroptosis in NPCs

**FIGURE 4:**
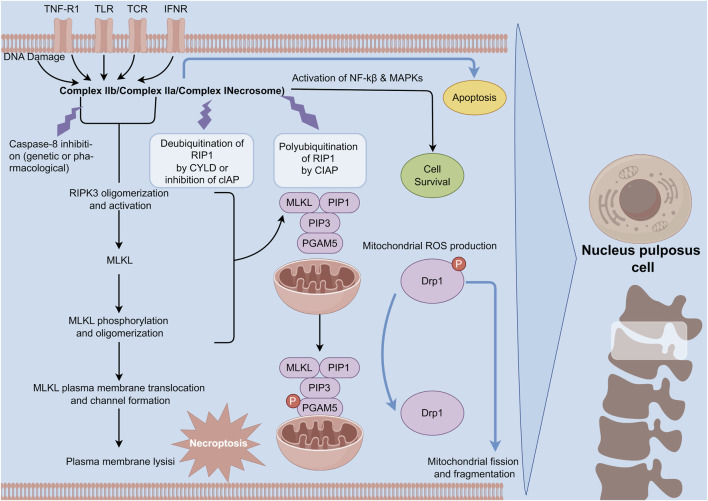
Mechanism of necroptosis in NPCs. Necroptosis is initiated by death receptor ligands. Upon the activation of upstream RIPK1, the activated RIPK1 is incorporated into the complex consisting of FADD, caspase-8, and caspase-10. In the circumstance of the absence of caspase-8 activity, RIPK1 enlists and phosphorylates RIPK3 to establish the pro-necrotic RIP1-RIP3 complex. Subsequently, these complex recruits and phosphorylates MLKL, which is conducive to the formation of a deleterious structure and programmed necrosis.

Unlike apoptosis, necroptosis—a form of regulated cell death characterized by necrosis—promotes inflammation. This process of necroptosis in NPC occurs in the context of compression-induced IVDD (Yurube et al., 2014). Research indicates that the expression levels of RIPK1, RIPK3, and MLKL, which are crucial proteins in necroptosis, are elevated under conditions of disc compression. This upregulation mediates necroptosis in NPC (Chen et al., 2017a). Myeloid differentiation primary response 88 (MyD88), a signaling molecule involved in innate immunity, also contributes to the necroptosis of NPC during IVDD (Bonnert et al., 1997). Fan et al. (2022) demonstrated that the levels of RIP3 and MLKL are elevated in NPC of degenerated discs compared to those in normal discs. Their cell experiments confirmed that inhibiting MyD88 reduces necrosis in compromised NPC (Fan et al., 2022) (Figure 4).

In addition, several factors associated with necroptosis have been confirmed to play a role in the progression of IVDD. Cao C. et al. (2022) demonstrated that pro-inflammatory factors, such as TNF and IL-1β, increase the expression of necroptosis related molecules, including RIPK1, RIPK3, and MLKL, thereby promoting NP cell death. In the experiments conducted by Hu et al. (2020); Lin et al. (2017), the upregulation of molecules such as HSP90 and Drp1 during compression promoted necroptosis of NPC. Furthermore, both the HSP90 inhibitor BIIB021 and the Drp1 inhibitor Minv-1 attenuated compression induced NPC death (Hu et al., 2020; Lin et al., 2017). In addition, ERS related proteins such as CHOP, GRP78, and PERK were found to be elevated in NPC undergoing IVDD (Lin et al., 2021). Hydrogen peroxide (H_2_O_2_) has been demonstrated to induce necroptosis in rat NPC (Shi et al., 2022).

These findings suggest that necroptosis plays a crucial role in the progression of IVDD. Despite the limited number of current studies, the detailed processes involving multiple signaling pathways and molecular mechanisms remain unclear. Further extensive research is needed to explore the underlying mechanisms of necroptosis in the context of IVD. Nonetheless, necroptosis represents a potential target for IVDD treatment.

### 3.3 Pyroptosis in NPCs

**FIGURE 5:**
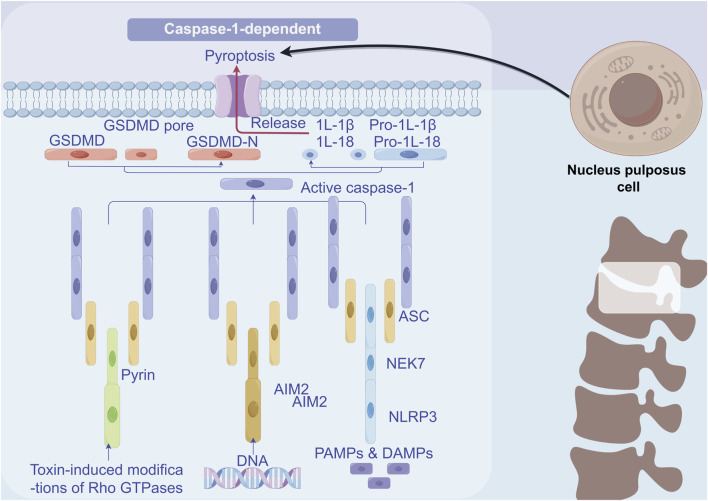
Mechanism of pyroptosis in NPCs. Inflammatory factors IL1β and TNF-α activate the NF-κB signalling pathway, thus inducing an inflammatory response. The mitochondrial autophagy pathway becomes activated. Moreover, ROS activation prompts the activation of NLRP3 inflammatory vesicles, which launches the key executor protein for pyroptosis, GSDMD, and ultimately leads to pyroptosis.

Pyroptosis, a mode of cell death mediated by inflammasome complexes, depends on the activation of caspase-1 and is characterized by a robust inflammatory response (Shi et al., 2015). Moreover, the cleavage of gasdermin D (GSDMD) results in the release of the GSDMD-N fragment, which subsequently activates caspase-1, leading to membrane pore formation and cell death (Sborgi et al., 2016). Numerous studies have investigated the role of pyroptosis in IVDD. Chen et al. found that the expression levels of NLRP3, caspase-1, and IL-1β were significantly elevated in the IVDD group compared to the normal group. The excessive activation of NLRP3 inflammasomes resulted in the overproduction of downstream IL-1β, which, as noted earlier, is implicated in the pathogenesis of human IVDD (Chen et al., 2015). Zhang et al. discovered that NLRP3 was activated during the pyroptosis of NPC in an IVDD mouse model (Zhang et al., 2020). Fu et al. demonstrated through animal experiments that abnormal mechanical loading of the spinal cord can induce pyroptosis and promote IVDD (Fu et al., 2021). Additionally, two other studies have shown that caspase-1 inhibitors can effectively delay the progression of IVDD when used with NLRP3 inflammasome inhibitors (Zhang et al., 2022; Xing et al., 2021) (Figure 5).

In conclusion, pyroptosis contributes to IVDD. Although the complete molecular mechanisms remain unclear, targeting pyroptosis represents a promising therapeutic direction for IVDD.

### 3.4 PANoptosis mechanisms in NPCs

PANoptosis is unique in that it is not merely a superposition of pyroptosis, apoptosis, and necrosis, but rather a novel cell death mechanism formed through their interaction and cross regulation. This mechanism has significant effects on cell function and fate (Cai et al., 2023). For example, Camilli et al. Demonstrates that nuclear export inhibitors (Selinexor, Eltanexor) induce PANoptosis in cancer models. These insights could parallel therapeutic approaches for IVDD (Camilli et al., 2023).

**FIGURE 6:**
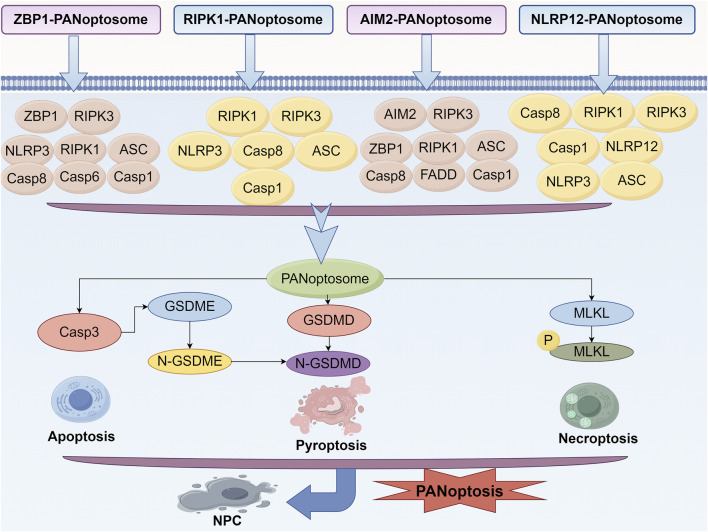
Mechanism of PANoptosis in NPCs. Signaling pathways of ZBP1-PANoptosome, RIPK1-PANoptosome, AIM2-PANoptosome and NLRP12-PANoptosome.

Although the exact mechanism of PANoptosis in NPCs is still in the early stage of investigation and little is reported in the literature, PANoptosis in NPCs plays a crucial role in regulating intervertebral disc function, particularly in the context of IVDD. As shown in Figure 6, reveals its effect in the process of PANoptosis in IVDD in main molecular mechanism of NPCs. Specifically, Zhou et al. successfully identified seven hub PANoptosis genes associated with vertebral IVDD using bioinformatics analysis and experimental validation (Zhou et al., 2024). Stimuli such as hydrogen peroxide (TBHP) can affect the expression of key proteins related to PANoptosis in NPCs, ultimately resulting in cell death and dysfunction (Chen et al., 2024). Kongensin A, a natural product, has been shown to inhibit PANoptosis, potentially by upregulating TAK1 expression. This mechanism may help maintain mitochondrial REDOX balance and protect the function of NPC (Chen et al., 2024). PANoptosis in NPC is influenced by various factors, including circular RNA (circRNA), which plays a significant role in regulating NPC function and PANoptosis. Specifically, circ_0004354 has been shown to promote the inflammatory response and apoptosis of NPCs by modulating the miR-345-3p-FAF1/TP73 axis, thereby accelerating the progression of IVDD (Li et al., 2022). Besides, the latest research indicates that PANoptosis is a mechanism driver of chronic inflammation in allergic bronchopulmonary aspergillosis. This is similar to the chronic inflammatory state in IVDD (Smallwood et al., 2024). In addition, activation of PANoptosis in a mouse model of cadmium exposure, which is similar to mechanical/oxidative stress of intervertebral discs, is a novel approach for IVDD studies (Camilli et al., 2024).

Therefore, the regulation of PANoptosis not only affects the survival of NPC but may also influence the overall health of the intervertebral disc. Further investigation into the mechanisms of PANoptosis in IVDD will provide a theoretical foundation and new insights for the treatment of IVDD.

## 4 Potential treatment strategies for PANoptosis in IVDD

PANoptosis involves multiple stages and contributes to the death of NPC. Therefore, inhibiting its progression in NPCs may present a potential therapeutic strategy for IVDD. For instance, targeting key molecules in the PANoptosis process, such as IL-2, Fas/FasL, and caspases, offers a promising treatment approach for IVDD, which will not be elaborated on here.

In addition to the previously mentioned molecules, many others warrant further investigation. For example, He et al. demonstrated that melatonin can attenuate oxidative stress induced apoptosis in NPC, suggesting it could be a promising therapeutic option for IVDD (He et al., 2018). Wang et al. demonstrated that dysregulated miR-155 promotes Fas mediated apoptosis in human NPC, suggesting a potential therapeutic role for miR-155 (Wang H. Q. et al., 2011). Cui et al. subsequently verified that the non-coding RNA MAGI2-AS3 is involved in regulating FasL expression in NPC (Cui et al., 2020). Additionally, it has been confirmed that lysyl oxidase (LOX) exhibits an anti-apoptotic effect in TNFα treated rat NP cells and may serve as a promising agent for the treatment of IVDD (Zhao R. et al., 2020).

In addition, bone marrow derived MSCS (BMSC) (Chen et al., 2017b),the mitochondria‐targeted anti‐oxidant MitoQ (Kang et al., 2020),allicin (Xiang et al., 2020), Sirtuin 3 (Song et al., 2018), cortistatin (Zhao Y. et al., 2020), Recombinant human SIRT1 (Miyazaki et al., 2015), Pyrroloquinoline quinone (Yang et al., 2015), Pramlintide (Wu et al., 2018) have also been confirmed to be involved in NPC death and are considered potential treatment options for IVDD.

Based on the existing studies, we have compiled PANoptisis modulators mentioned above, as shown in Table 3, which may provide new ideas for the treatment of PANoptisis in IVDD. Most of the research has been conducted using animal models, and there is a lack of sufficient clinical evidence to prove the feasibility and effectiveness of these treatments in clinical applications. However, these studies can still provide valuable insights for treating IVDD through PANoptosis.

**TABLE 3:** PANoptosis modulators in IVDD.

Therapeutic molecule	Target	Models	PANoptosis inducer/inhibitor	Ref
Kongensin A	TAK1 kinase inhibition	NP cells, rat	Inhibitor	Chen et al. (2024)
Circ_0004354	miR-345-3p-FAF1/TP73 axis	NP cells, human	Inducer	Li et al. (2022)
Melatonin	SIRT1/SIRT3 activation; ROS suppression	NP cells, rat	Inhibitor	He et al. (2018)
miR-155	cGAS-STING pathway activation	NP cells, human	Inducer	Wang H. Q. et al. (2011)
RNA MAGI2-AS3	FasL	NP cells, human	Inducer	Cui et al. (2020)
Lysyl oxidase	MLKL phosphorylation inhibition	NP cells, rat	Inhibitor	Zhao R. et al. (2020)
Bone marrow derived MSCS	IL-10 secretion; NLRP3/Caspase-1 suppression	NP cells, rat	Inhibitor	Chen et al. (2017b)
MitoQ	Mitochondrial ROS	NP cells, human	Inducer	Kang et al. (2020)
Allicin	Gasdermin D pore formation	NP cells, human	Inducer	Xiang et al. (2020)
Sirtuin 3	Mitochondrial antioxidant enhancement	NP cells, human	Inhibitor	Song et al. (2018)
Cortistatin	NF-κB/MAPK pathway inhibition	NP cells, mice	Inhibitor	Zhao Y. et al. (2020)
Recombinant human SIRT1	NLRP3 deacetylation	NP cells, human	Inhibitor	Miyazaki et al. (2015)
Pyrroloquinoline quinone	Nrf2 pathway activation	NP cells, rat	Inhibitor	Yang et al. (2015)
Pramlintide	Amylin receptor signaling modulation	NP cells, human	Inhibitor	Wu et al. (2018)

## 5 Conclusions and perspectives

Recently, research of cell death has gradually expanded from traditional apoptosis, pyroptosis, and necroptosis to more complex mechanisms. PANoptosis, as a new mode of cell death, is increasingly showing its important role in IVDD. By conducting an in-depth analysis of the mechanism of PANoptosis, we can gain a more comprehensive understanding of the cell death process and its influencing factors in IVDD (Pandeya and Kanneganti, 2024).

Various research findings indicate that PANoptosis is involved not only in PCD but also in inflammatory responses and alterations in the cellular microenvironment. (Cai et al., 2023; Chen et al., 2024). The complexity of this mechanism necessitates considering the interplay of multiple factors when developing treatment strategies. Consequently, a comprehensive study of apoptosis, pyroptosis, and necroptosis will support the development of more effective intervention strategies. Coordinated investigations of these cell death pathways could aid in defining the multiple mechanisms of disc degeneration, thereby enhancing therapeutic outcomes.

Exploring the interrelation between PANoptosis and other cell death pathways in depth will be an important research direction for future studies. This exploration could not only uncover new biomarkers but also reveal novel therapeutic targets. Additionally, from a clinical application perspective, it is essential to investigate how to translate the research findings of this mechanism into practical treatment options to more effectively address IDD related diseases.

In summary, PANoptosis, as a key mechanism linking various cell death pathways, offers a new direction for the research and treatment of IVDD. Future studies should continue to advance in this field to develop more effective treatment options for patients with IVDD.
